# The Effect of *Clostridium butyricum* on Gut Microbiota, Immune Response and Intestinal Barrier Function During the Development of Necrotic Enteritis in Chickens

**DOI:** 10.3389/fmicb.2019.02309

**Published:** 2019-10-11

**Authors:** Ting Huang, Xin-Yu Peng, Biao Gao, Qi-Lin Wei, Rong Xiang, Ming-Gui Yuan, Zhi-Hong Xu

**Affiliations:** ^1^Institute of Animal Health, Guangdong Academy of Agricultural Sciences, Guangzhou, China; ^2^Key Laboratory of Livestock Disease Prevention of Guangdong Province, Guangzhou, China; ^3^Scientific Observation and Experiment Station of Veterinary Drugs and Diagnostic Techniques of Guangdong Province, Ministry of Agriculture, Guangzhou, China; ^4^Chinese Traditional Medicine Engineering Technology Research Center of Guangdong Province, Guangzhou, China

**Keywords:** *Clostridium perfringens*, *Clostridium butyricum*, necrotic enteritis, gut microbiota, mucosal immunity, intestine barrier

## Abstract

Necrotic enteritis (NE) causes huge economic losses to the poultry industry. Probiotics are used as potential alternatives to antibiotics to prevent NE. It is known that *Clostridium butyricum* can act as a probiotic that can prevent infection. However, whether or not it exerts a beneficial effect on NE in chickens remains elusive. Therefore, we investigated the impact of *C. butyricum* on immune response and intestinal microbiota during the development of NE in chickens, including experimental stages with basal diets, high-fishmeal-supplementation diets, and *Clostridium perfringens* challenge. Chickens were divided into two groups from day 1 to day 20: one group had its diet supplemented with *C. butyricum* supplementation and one did not. At day 20, the chickens were divided into four groups: *C. perfringens* challenged and unchallenged chickens with and without *C. butyricum* supplementation. All groups were fed a basal diet for 13 days and thereafter a basal diet with 50% fishmeal from day 14 to 24. Chickens were infected with *C. perfringens* from day 21 to 23. At days 13, 20 and 24, samples were collected for analysis of the relative expression of immune response and intestinal mucosa barrier-related genes and intestinal microbes. The results show that *C. butyricum* can inhibit the increase in IL-17A gene expression and the reduction in Claudin-1 gene induced-expression caused by *C. perfringens* challenge. Moreover, *C. butyricum* was found to increase the expression of anti-inflammatory IL-10 in infected chickens. Although *C. butyricum* was found to have a significant beneficial effect on the structure of intestinal bacteria in the basal diet groups and decrease the abundance of *C. perfringens* in the gut, it did not significantly affect the occurrence of intestinal lesions and did not significantly correct the shift in gut bacterial composition post *C. perfringens* infection. In conclusion, although *C. butyricum* promotes the expression of anti-inflammatory and tight junction protein genes and inhibits pro-inflammatory genes in *C. perfringens-*challenged chickens, it is not adequate to improve the structure of intestinal microbiota in NE chickens. Therefore, more effective schemes of *C. butyricum* supplementation to prevent and treat NE in chickens need to be identified.

## Introduction

Necrotic enteritis (NE), which is caused by *Clostridium perfringens* in broiler chickens, is a common disease in the poultry industry, resulting in huge financial losses every year. It is estimated that NE causes losses of more than $US 6 billion per year and poses a substantial risk to public health (Wade Ben, 2015). However, *C. perfringens* infection alone does not cause clinical NE (Olkowski et al., 2006; Shojadoost et al., 2012). Predisposing factors, such as a high-fishmeal-supplementation diet or/and *Eimeria* coinfection, combined with *C. perfringens* infection, are required to induce NE in chickens (Wu et al., 2010, 2011; Fernandes da Costa et al., 2013). It has been reported that high-fishmeal diets that include high available nutrition cause an increase in *C. perfringens-*associated lesions in the chicken gut (Drew et al., 2004). We used a high-fishmeal-supplementation diet and *C. perfringens* challenge to induce NE in chickens. According to the findings of previous studies (Wu et al., 2010; Fernandes da Costa et al., 2013), the induction of NE in this study was divided into three main stages: (i) 1-13-day-old chickens on a basal diet, (ii) 14-20-day-old chickens on a high-fishmeal-supplementation diet, (iii) 21-24-day-old chickens on a high-fishmeal-supplementation diet and *C. perfringens* challenge.

Usually, NE is prevented through the use of antibiotics. However, many countries have restricted the use of antibiotics owing to the emergence of resistant strains, disruption of the host gut microbiota, and residual contamination of products and the environment. Probiotics, essential oils and organic acids are among the potential alternatives to antibiotics (Allaart et al., 2013). *Clostridium butyricum* is a butyric acid-producing gram-positive anaerobe that commonly exists in the intestines of humans and animals. In chickens, butyric acid inhibits pathogens and improves intestine barrier function (Meimandipour et al., 2010; Zhang et al., 2011), benefits intestinal microbiota, and stimulates immune factors (Kong et al., 2011; Zhang et al., 2011; Gao et al., 2012b; Yang et al., 2012). *C. butyricum* has been used as a probiotic that decreases the clinical signs of several diseases such as inflammatory bowel disease (IBD) and antibiotic-associated diarrhea (Seki et al., 2003; Yasueda et al., 2016). Moreover, previous studies have shown that *C. butyricum* could be a suitable alternative for reducing colonization by pathogens such as *Salmonella* and *Escherichia coli* (Zhang et al., 2016; Zhao et al., 2017).

However, the effect of *C. butyricum* on NE in chickens remains to be elucidated. Therefore, we aimed to explore the effects of *C. butyricum* on gut microbiota, immune response, and intestinal barrier function-related genes during the development of NE in chickens.

## Materials and Methods

### Ethics Statement

This study was carried out in accordance with the principles of the Basel Declaration and the recommendations of the Institutional Animal Care and Use Committee guidelines, Guangdong Academy of Agricultural Sciences. The study protocol was approved by the Guangdong Academy of Agricultural Sciences (No. SYXK (yue)-20180108).

### Bacterial Strains

The pathogenic *C. perfringens* cpnetBF carrying the *netB* gene was isolated from the intestines of broiler chickens with NE in Guangdong Province, China (Huang et al., 2018). The strain was stored at −80°C in fluid thioglycollate broth (FT, Beckson, Dickinson, and Company) supplemented with 30% glycerol. *C. butyricum* YH 018 with a count of 1 × 10^9^ cfu/g was obtained from Yihao Biotic Co., Ltd., China. The phylogenetic relationships based on 16S rRNA gene sequences are shown in Supplementary Figure S1. The concatenated sequences were aligned by MEGA 6 to infer a maximum-likelihood tree. *C. butyricum* YH 018 is clustered with type strains of species of the *C. butyricum* phylogroup including *C. butyricum* strain JCM 1391T and ATCC 19398T.

### Animal Trial Design and Sample Collection

One-day-old Ross 308 commercial chickens (negative for *C. perfringens*) were purchased from a hatchery in Guangzhou City, Guangdong Province, China. The chickens were given food and water *ad libitum* and maintained in a 12 h light/12 h dark cycle. The animal trial was conducted at a poultry house located at the Institute of Animal Health, Guangdong Academy of Agricultural Sciences. A total of 120 chickens were randomly divided into two groups. All groups were fed an antibiotic-free basic chicken diet between the ages of 1 and 13 days (Supplementary Table S1) and then switched to a wheat-based diet containing 50% fishmeal from day 14 to 24. One group was supplemented with 1 × 10^9^ cfu of *C. butyricum*/kg between the ages of 1 and 24 days. At an age of 20 days, each of the above-mentioned groups of chickens was further divided into two groups. From the ages of 21 days to 23 days, the *C. butyricum* supplemented and non-supplemented groups were challenged with *C. perfringens* cpnetBF-inoculated feed, as previously described (Stanley et al., 2012).

**FIGURE 1:**
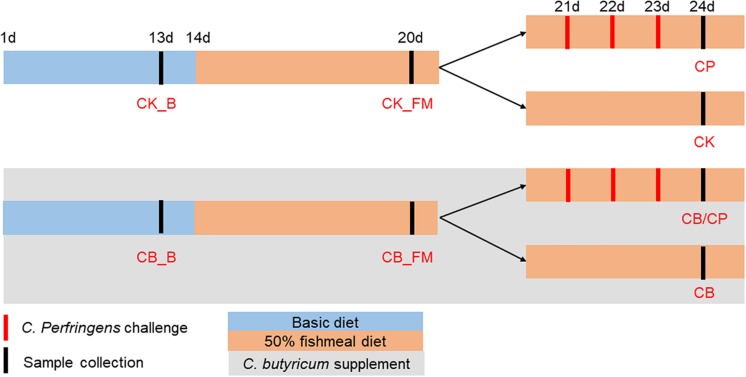
Experimental design. CB_B (*n* = 60): *C. butyricum* supplemented chickens on a basal diet; CK_B (*n* = 60): *C. butyricum* non-supplemented chickens on a basal diet; CB_FM (*n* = 48): *C. butyricum* supplemented chickens on a high-fishmeal diet; CK_FM (*n* = 48): *C. butyricum* non-supplemented chickens on a high-fishmeal diet; CB/CP (*n* = 18): *C. perfringens-*challenged chickens with *C. butyricum* supplementation; CB (*n* = 18): *C. perfringens-*unchallenged chickens with *C. butyricum* supplementation; CP (*n* = 18): *C. perfringens*-challenged chickens without *C. butyricum* supplementation; CK (*n* = 18): *C. perfringens*-unchallenged chickens without *C. butyricum* supplementation.

Chickens from each group were euthanized using cervical dislocation at an age of either 13, 20, or 24 days. Twelve replicates each of the jejunum-ileum contents of groups CB_B, CK_B, CB_FM, and CK_FM and six replicates each of the jejunum-ileum contents of groups CB/CP, CB, CP, and CK groups were collected for DNA extraction. The 12 ileal tissue replicates in each of the CB_B, CK_B, CB_FM and CK_FM groups and the six ileal tissue replicates in each of the CB/CP, CB, CP and CK groups were collected from a distance of 3 cm distal to the Meckel’s diverticulum and were immediately frozen in liquid nitrogen. The *C. butyricum* supplemented group was named CB_B (*n* = 60) and CB_FM (*n* = 48) at ages of 13 and 20 days, respectively, while the *C. butyricum* non-supplemented group was named CK_B (*n* = 60) and CK_FM (*n* = 48) at ages of 13 and 20, respectively. At an age of 24 days, the names of the four groups were as follows: CB/CP (*n* = 18): *C. perfringens-*challenged chickens with *C. butyricum* supplementation, CB (*n* = 18): *C. perfringens-*unchallenged chickens with *C. butyricum* supplementation, CP (*n* = 18): *C. perfringens*-challenged chickens without *C. butyricum* supplementation, and CK (*n* = 18): *C. perfringens-*unchallenged chickens without *C. butyricum* supplementation, as shown in Figure 1.

### Intestinal Lesion Score

At an age of 24 days, the lesions of the small intestine (duodenum to ileum) were scored on the basis of 10 replicates each from the four groups at the challenge stage, as previously described (Keyburn et al., 2006): 0, no gross lesions; 1, thin or friable walls; 2, focal necrosis or ulceration (one to five foci); 3, focal necrosis or ulceration (six to 15 foci); 4, focal necrosis or ulceration (16 or more foci); 5, patches of necrosis 2–3 cm long; 6, diffused necrosis typical of field cases.

### DNA Extraction and the Abundance of *C. perfringen*s

DNA from the jejunum-ileum contents was extracted using a QIAamp Fast DNA Stool Mini Kit (Qiagen, Valencia, CA, United States), according to the manufacturer’s instructions. Total DNA was quantified on a NanoDrop^®^ ND-2000 UV spectrophotometer (NanoDrop Technologies, Wilmington, DE). Only DNA samples with an A260/A280 value >1.7 and A260/A230 value >1.8 were used for further analysis (Li et al., 2014). The extracts were stored at −20°C until used (Singh et al., 2015).

Quantitative PCR reaction was performed on the intestinal DNA template, in triplicate, using an SYBR Premix Ex Taq (Takara Bio, Otsushi, Japan). The expression of the *C. perfringens* genes was normalized using the 16S rRNA gene (Buffie et al., 2015). The relative abundance of *C. perfringens* in the intestine was calculated based on the value of the 16S rRNA gene using the 2^–Δ^ ^Ct^ method. The primers used for the qPCR of *C. perfringens* and 16S rRNA gene are shown in Supplementary Table S2.

### 16s rRNA Gene Sequencing

The gene-specific sequences for the 16S V3-V4 region were amplified using primers 338F (5′- ACTCCTACGGGAGGCA GCAG-3′) and 806R (5′-GGACTACHVGGGTWTCTAAT-3′) using a thermocycler PCR system (GeneAmp 9700, ABI, United States). The PCR conditions were as follows: 3 min of denaturation at 95°C, 27 cycles of 30 s at 95°C, 30s for annealing at 55°C, 45s for elongation at 72°C, and a final extension step at 72°C for 10 min. PCR reactions were performed in triplicate using a 20-μL mixture containing 4 μL of 5 × FastPfu Buffer, 2 μL of 2.5 mM dNTPs, 0.8 μL of each primer (5 μM), 0.4 μL of FastPfu Polymerase, and 10 ng of template DNA. The resulting PCR products were extracted from the 2% agarose gel and further purified using the AxyPrep DNA Gel Extraction Kit (Axygen Biosciences, Union City, CA, United States) and quantified using a QuantiFluor^TM^-ST (Promega, United States) according to the manufacturer’s protocol. Bar-coded V3-V4 amplicons were sequenced using the 2 × 300 paired-end method by Illumina MiSeq with a 7-cycle index read. Sequence processing was performed using QIIME 2 to process the data. Operational taxonomic units (OTUs) were clustered using a 97% similarity cutoff in UPARSE (version 7.1^1^), and chimeric sequences were identified and removed using UCHIME. The taxonomy of each 16S rRNA gene sequence was analyzed using an RDP Classifier algorithm^2^ against the Silva (SSU123) 16S rRNA database using a confidence threshold of 70%. Simpson and abundance-based coverage (ACE) indices were calculated for each time point to represent taxonomic alpha diversity. The Venn diagrams were calculated using R software. The microbial community structures of the different samples were compared using Bray-Curtis dissimilarity. Principal coordinate analysis (PCoA) was conducted to assess the relationships among the different groups. The linear discriminate analysis (LDA) effect size (LEfse) using the non-parametric factorial Kruskal-Wallis (KW) sum-rank test was used to identify significant differences in the relative abundance of bacteria between two groups. The functional potentials of the intestinal microbiotas were predicted using the Phylogenetic Investigation of Communities by Reconstruction of Unobserved States (PICRUSt).

### RNA Isolation and RT-qPCR of the Immune Genes

Total RNA was isolated from ileal tissue using the EZNA^®^ Total RNA Isolation Kit (Omega Bio-Tek) according to the manufacturer’s instructions. RNA was eluted in DEPC-treated water, quantified using a NanoDrop ND-2000 UV spectrophotometer (NanoDrop^®^ Technologies, Wilmington, DE, United States), and stored at −80°C until further use.

Reverse transcription of the isolated RNA was performed using an M-MLV First Strand cDNA Synthesis Kit (Omega Bio-Tek) following the manufacturer’s instructions. RT-qPCR was performed with cDNA temple in triplicate using SYBR Premix Ex Taq (Takara Bio, Otsushi, Japan). The expressions of TLR2, IL-10, TNF, IL-1β, NF-κB, IL-17A, Claudin-1, Claudin-2, Occduin1, and IgA genes were normalized to glyceraldehyde-3-phosphate dehydrogenase (GAPDH) (Zhang et al., 2017). The relative expression was calculated based on the expression of GAPDH, using the 2^–Δ^ ^Ct^ method. The primers used for RT-qPCR are shown in Supplementary Table S2.

### Statistical Analysis

Statistical analysis of the relative expression of mRNA, the relative abundance of *C. perfringens* and the alpha diversity indices of Simpson and ACE were performed using ANOVA. A *p-*value of <0.05 was considered to be statistically significant. For species abundance, comparisons were made using the Kruskal-Wallis test with Benjamini-Hochberg *p-*value correction.

## Results

### *Clostridium perfringens* Colonization

**FIGURE 2:**
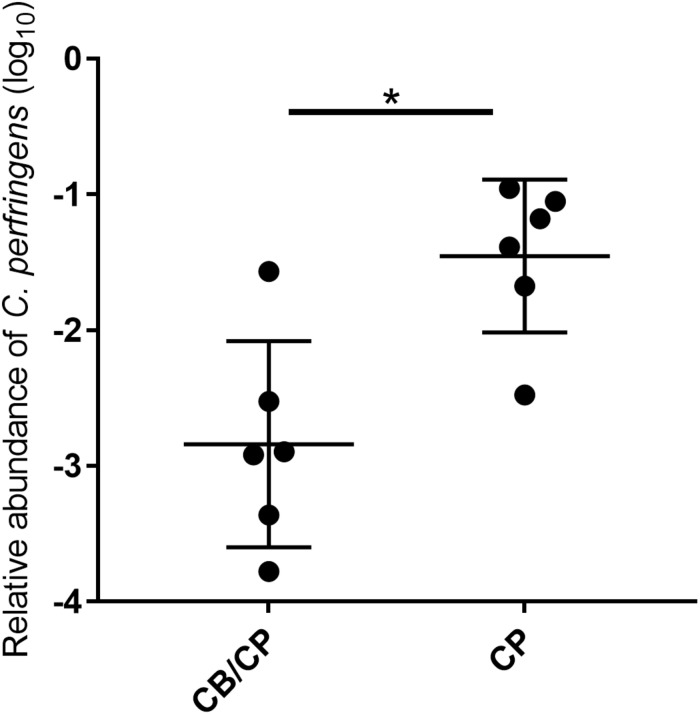
Relative abundance of *C. perfringens* in the jejunum-ileum lumen post 1-day challenge. All chickens tested negative for *C. perfringens* until experimentally challenged, and the unchallenged groups tested negative for *C. perfringens* throughout the study. ^∗^*p* < 0.05, measured using ANOVA; *n* = 6.

All chickens were negative for *C. perfringens* until the challenge, and the unchallenged groups remained negative throughout this study. In the challenged groups, the relative abundance of *C. perfringens* in chickens with *C. butyricum* supplementation was significantly lower than that of *C. butyricum* non-supplemented chickens (*p* < 0.05, Figure 2).

### Intestinal Lesion Score

**FIGURE 3:**
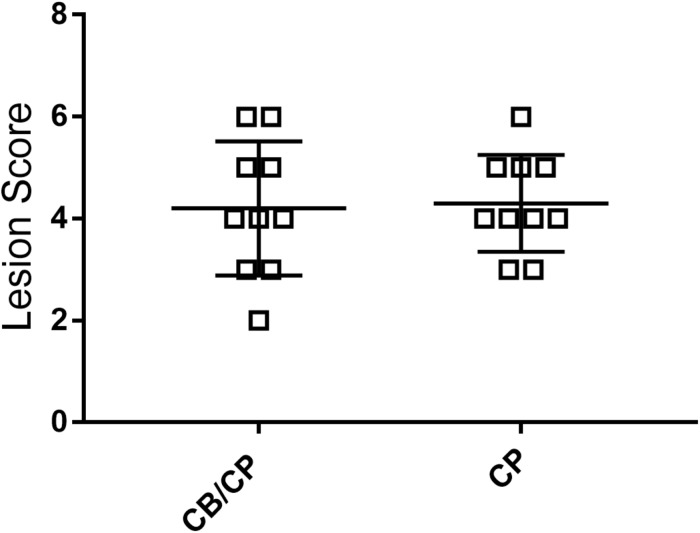
Intestinal lesion scores of chickens after *C. perfringens* infection. Measured using ANOVA; *n* = 10. No intestinal lesion was found in the unchallenged groups.

No intestinal lesions were observed in unchallenged chickens. The infection-induced intestinal lesion score was not influenced by the presence or absence of *C. butyricum* supplementation (*p* > 0.05, Figure 3).

### Expression of Immune Response Genes

**FIGURE 4:**
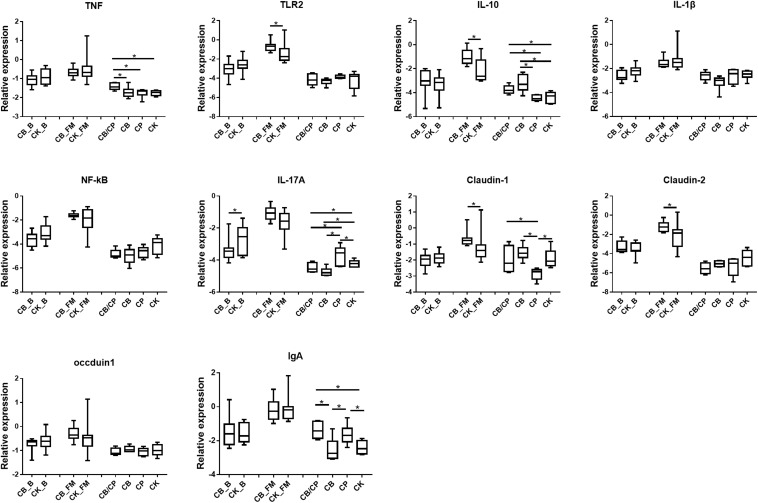
Relative expression of immune and intestinal mucosa barrier-related genes in the ileum. Relative gene expression is represented as log_10_ 2^–Δ^ ^Ct^. Significant differences are indicated by an asterisk (ANOVA, ^∗^*p* < 0.05). CB_B, CK_B, CB_FM and CK_FM: *n* = 12; CB/CP, CB, CP and CK: *n* = 6.

The expressions of the immune and intestinal barrier-related genes detected in the different groups are shown in Figure 4. In the basal diet chickens, only IL-17A expression was significantly different between the *C. butyricum* supplemented and non- supplemented groups. The relative expressions of TLR2, IL-10, Claudin-1, and Claudin-2 were significantly higher in the CB_FM group than in the CK_FM group (*p* < 0.05). Compared with the other three groups, *C. perfringens* infection induced the highest expression of TNF in the CB/CP group. The expression of IL-10 showed no significant difference between the CP and CK group; however, it was significantly higher in the CB/CP group than in the CP group. The relative expression of IL-17A increased significantly in the CP group compared in with the CK group and significantly decreased in the CB/CP and CB groups compared in with the CP and CK groups. Conversely, the expression of Claudin-1 significantly decreased in the CP group compared with in the CK group and increased significantly in the CB/CP group compared in with the CP group (*p* < 0.05). The *C. perfringens* challenge significantly increased the relative expression of IgA, but *C. butyricum* had no effect on the level of IgA. There were no significant differences between the relative expressions of the IL-1β, NF-κB, and occludin-1 genes of the groups at different stages.

### Intestinal Bacterial Diversity Analysis

**FIGURE 5:**
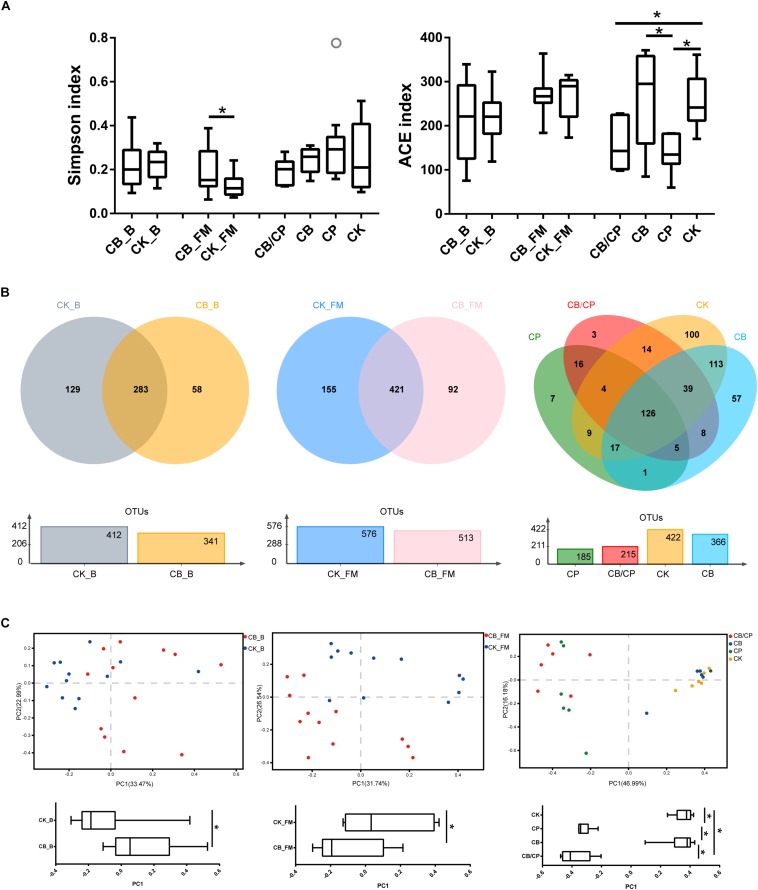
Diversity of the intestinal bacteria community at different stages. **(A)** Alpha diversity was estimated using Simpson and ACE indices values. ^∗^*p* < 0.05, measured using ANOVA. **(B)** Unique and shared intestinal OTUs shown in Venn diagrams. **(C)** PCoA plot of Bray-Curtis indices for the gut microbiota. PC1 comparisons were made using ANOVA. ^∗^*p* < 0.05. CB_B, CK_B, CB_FM and CK_FM: *n* = 12; CB/CP, CB, CP and CK: *n* = 6.

A total of 856,602, 1,034,832, and 916,190 high-quality 16S rRNA sequences were obtained from 13-, 20-, and 24-day-old intestinal content samples, respectively. The Simpson and ACE indices were used as measures of the alpha diversity (evenness and richness) of the gut microbiota (Figure 5A). In the basal diet groups, no significant differences were found between the ACE and Simpson index values of the CB_B and CK_B groups. During the stage of high-fishmeal supplementation, *C. butyricum* significantly increased the Simpson indices in the CB_FM group compared with in the non-supplemented CK_FM group (*p* < 0.05). Infection by *C. perfringens* significantly decreased the ACE indices compared with the unchallenged groups, CB and CK (*p* < 0.05). However, there was no significant difference between the CB/CP and CP groups.

A Venn diagram reveals the shared and specific OTUs of the different groups (Figure 5B). Dietary *C. butyricum* decreased the number of unique OTUs in the CB_B (58) and CB_FM (92) groups compared with the CK_B (129) and CK_FM (155) groups, respectively. At the stage of *C. perfringens* challenge, the four groups shared 126 OTUs. In addition, compared with the CB/CP group, the CP group had a less unique OTU and shared less OTUs with the CK group.

Principal coordinate analysis (PCoA) of Bray-Curtis indices demonstrates the diversity of bacteria among the different groups (Figure 5C). Before *C. perfringens* challenge, the gut bacterial structure of the chickens with *C. butyricum* supplementation (CB_B and CB_FM) was found to be distinct from that of the groups without *C. butyricum* (CK_B and CK_FM). After *C. perfringens* challenge, the samples from the CB/CP and CP groups were found to be distinct from those of the CB and CK groups. However, the microbial structure of the CB/CP group was not significantly different from that of the CP group (*p* < 0.05).

### Structure and Composition of Intestinal Bacteria

**FIGURE 6:**
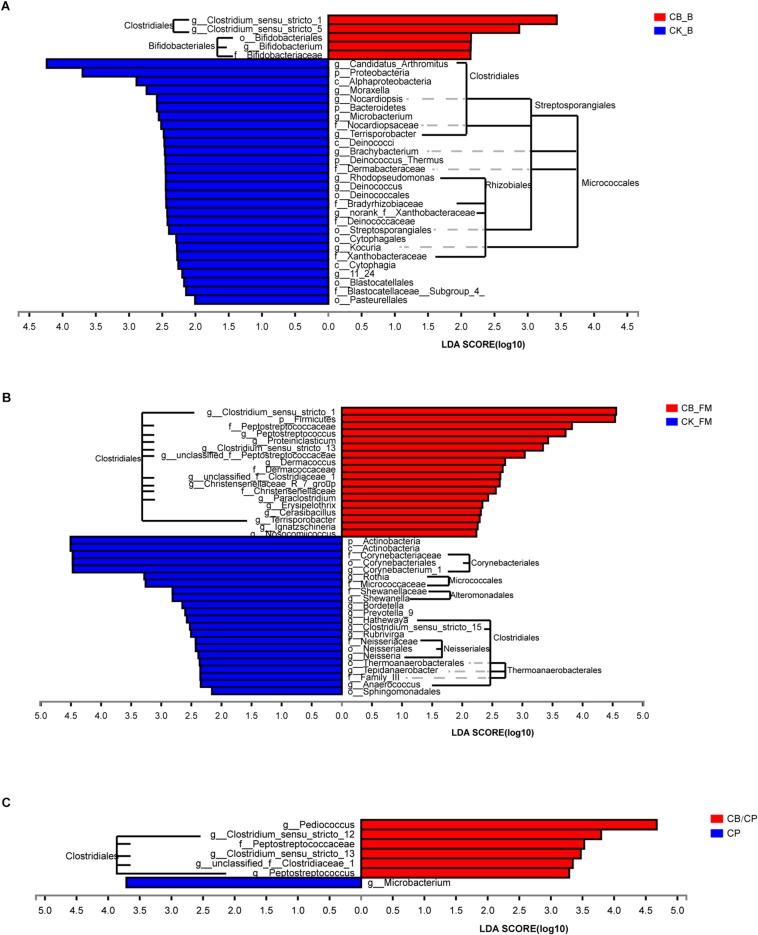
Differential enrichment of intestinal microbiota between groups measured using LEfSe (LDA score = 3). Bars represent significant abundance from phylum to genus between **(A)** the CB_B and CK_B groups, **(B)** the CB_FM and CK_FM groups, **(C)** and the CB/CP and CP groups. CB_B, CK_B, CB_FM and CK_FM: *n* = 12; CB/CP, CB, CP and CK: *n* = 6.

At the order level, the predominant microorganisms in the chicken intestine were *Lactobacillales* and *Clostridiales* (Figure 6 and Supplementary Figure S2). Following high-fishmeal supplementation, *Corynebacteriales* decreased dramatically in the CB_FM group compared with in the CK_FM group (Supplementary Figure S2). After *C. perfringens* challenge, *Clostridiales* significantly increased in the CP group compared with in the CK group, whereas *Bacillales* decreased. Moreover, these microbiotic changes were significantly alleviated in the CB/CP group. In addition, LEfSe showed that high-fishmeal supplementation specifically increased the overrepresented bacteria, *Clostridiales*, in the CB_FM group (Figure 6), while similar results were also noted in the CB/CP group. These observations reveal that dietary *C. butyricum* increases the relative proportion of *Clostridiales* at different stages of NE.

For further investigation of the composition of bacteria at different stages, the significance of differences in microbial species abundance was analyzed (Supplementary Table S3). *C. butyricum* increased significantly in the *C. butyricum-*supplemented groups, especially at the stage of high-fishmeal supplementation. Moreover, the abundance of *Lactobacillus*_sp._KC45b significantly increased and *Candidatus Arthromitus* and unclassified_g__*Brachybacterium* significantly decreased in the CB_B group compared with in the CK_B group. In the *C. perfringens-*challenged groups, CB/CP and CP, the abundance of *Lactobacillus salivarius* decreased significantly compared with in the CK group. Notably, the abundances of *Weissella thailandensis*, unclassified_g__*Weissella*, and *Pediococcus acidilactici* in the CB/CP group were higher than in the CK group. However, no significant differences between the CB/CP and CP groups or between the CB and CK groups were found using cutoff values of *p* < 0.05 and FDR < 0.1.

### Predicting the Function of Intestinal Bacteria

**FIGURE 7:**
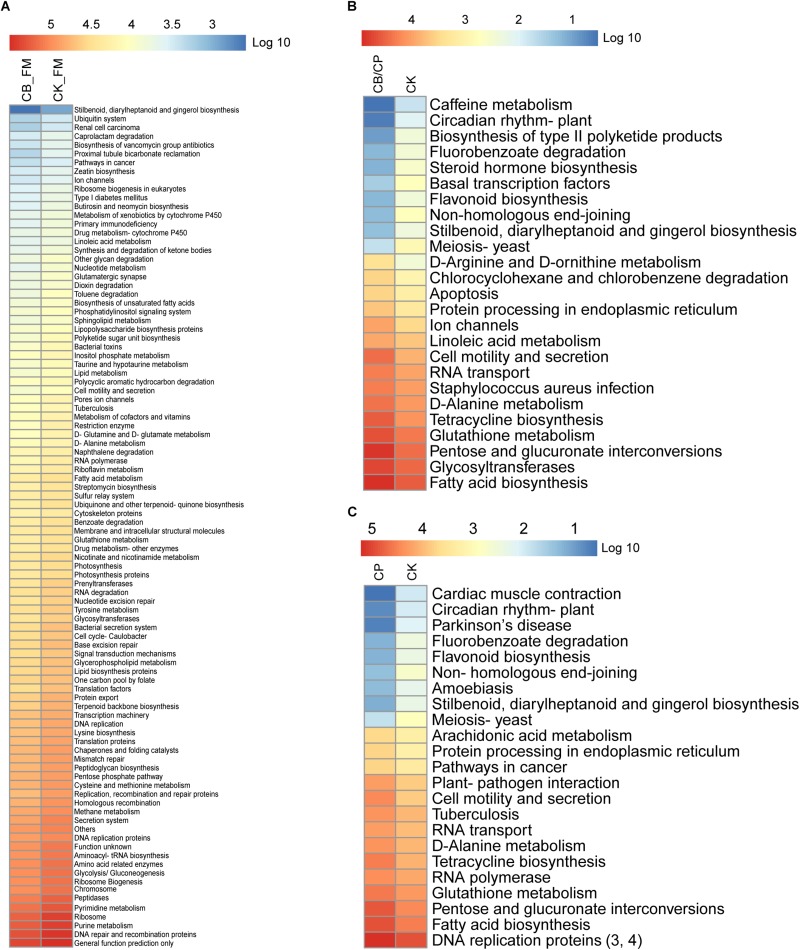
Heatmap of predicted KEGG pathways of the gut bacteria in each group. The gene copy numbers of each group are shown as mean values. The significant differences between two groups at different stages were measured using the Mann-Whitney U test with a cutoff of *p* < 0.05 and FDR < 0.1. No significant difference was found between the CB_B and CK_B groups, the CB/CP and CP groups, and the CB and CK groups. Comparisons between the CB/CP and CB groups and the CB and CP groups were not performed. CB_B, CK_B, CB_FM, and CK_FM: *n* = 12; CB/CP, CB, CP, and CK: *n* = 6. Comparisons between CB_FM and CK_FM groups, CB/CP and CK groups and CP and CK groups are presented in panel **(A)**, **(B)**, and **(C)** respectively.

The functions of the intestinal microbiota metagenome were predicted using PICRUSt from the Kyoto Encyclopedia of Genes and Genomes pathways (Figure 7). A total of 281 functions were detected in all of the groups. No significant differences were found between the CB_B and CK_B groups and the CB/CP and CP groups. However, there were significant differences (Mann-Whitney U test, *p* < 0.05, FDR < 0.1) between the CB_FM and CK_FM groups in 96 functions, comprising 34.16% of the total functions, including purine metabolism and pyrimidine metabolism. Moreover, there were 16 and 14 significant differences between the CB/CP and CK groups and the CP and CK groups, respectively, for example, fatty acid biosynthesis, pentose and glucuronate interconversions, and glutathione metabolism.

All raw sequences were submitted to the Sequence Read Archive database at NCBI (accession no. SRP159781).

## Discussion

Previous reports have shown that NE is caused by factors including a fishmeal diet, *Eimeria*, and *C. perfringens* and is associated with a shift in gut microbiota structures and inflammation (Stanley et al., 2012, 2014). *C. butyricum* is a probiotic that has been proven to alleviate certain infections. Hence, we investigated the effect of *C. butyricum* on gut bacterial community composition and immune factors during the development of the NE caused by a high-fishmeal diet and *C. perfringens* infection.

The immune system plays a critical role in fighting infections. It has been reported that dietary *C. butyricum* can promote the expression of Claudin and Occludin to maintain intestinal barrier function, activate the expression of NF-κB, TLR2, and IL-10 and decrease pro-inflammatory cytokines, IL-1β, and TNF expression to modulate inflammatory response (Gao et al., 2012b; Hayashi et al., 2013; Zhang et al., 2016; Chen et al., 2018; Li H. et al., 2018; Liu et al., 2018). However, other studies have reported that *C. butyricum* has no influence on the immune system of neonatal mice and pigs (Miao et al., 2018; Peeters et al., 2019). In line with this finding, in this study, during the basal diet stage, no significant differences were found between *C. butyricum* supplemented and non-supplemented groups in the expression of every intestinal barrier function and inflammatory gene apart from IL-17A. A previous study reported that the composition of the diet is associated with the efficacy of *C. butyricum*. A *C. butyricum* supplemented diet improves the immune response and promotes intestinal barrier function with a low digestibility diet, whereas *C. butyricum* has no effect with a highly digestible diet (Chen et al., 2018). Based on these findings, we speculate that intestinal barrier function and inflammatory genes are probably not affected by *C. butyricum* in the findings of this study due to the type of diet.

Intestinal dysbiosis can elevate the production of pro-inflammatory cytokines, IL-1β, TNF, and NF-κB, and *C. butyricum* can decrease the expression of these factors in piglets, suggesting that *C. butyricum* can alleviate the inflammatory response in piglets (Chen et al., 2018; Cao et al., 2019; Wang et al., 2019). Zhang et al. reported that *C. butyricum* promotes the expression of TNF in chickens with *E. coli* K88 challenge (Zhang et al., 2016). Similar results were obtained in the current study, where *C. butyricum* was found to increase the expression of TNF in response to *C. perfringens* infection. A TNF-induced inflammatory response is common in chickens infected with pathogens such as *Salmonella* (Feng et al., 2016). Similarly, the NetB toxin of *C. perfringens* increases the TNF mRNA level in the intestinal lymphocytes of chickens (Jang et al., 2012). However, in this study, the expressions of TNF and TRL2 were not affected by *C. perfringens* challenge, which is consistent with the results of previous studies (Lu et al., 2009; Du et al., 2016). The high expression of IL-17A is associated with the initiation of several inflammatory diseases (Kolls and Linden, 2004). As previously mentioned, the results of this study demonstrate that *C. perfringens* challenge can upregulate the expression of IL-17 (Fasina and Lillehoj, 2019). Previous research has shown that a decrease in IL-17A expression can ameliorate inflammation (Kanai et al., 2015). Various mechanisms have been proposed to explain the anti-inflammatory effects of probiotics on intestinal inflammation, one of which is suppression of the expression of IL-17 (Tanabe, 2013). At the same time, it has been proven that the intake of *Bacillus subtilis* by mice with inflammatory bowel disease can downregulate the expression of Il-17 in order to decrease intestinal damage (Gong et al., 2016). We observed that *C. butyricum* could inhibit the upregulation of IL-17A expression induced by *C. perfringens*. Moreover, *C. butyricum* increases the expression of anti-inflammatory IL-10 mRNA, which plays a vital role in preventing many inflammatory diseases (Gao et al., 2012a). For instance, *Bifidobacterium*-induced IL-10 production benefits patients with colitis (Imaoka et al., 2008). In the same manner, we found that *C. butyricum* increases the expression of IL-10 mRNA levels in *C. perfringens*-challenged chickens, a situation that may indicate the prevention of NE.

IgA is the predominant intestinal immunoglobulin that contributes to intestinal barrier function (Pabst, 2012). Previous studies have reported that dietary *C. butyricum* supplementation can promote the expression of IgA in rabbits, broiler chickens, and mice (Murayama et al., 1995; Yang et al., 2012; Liu et al., 2018). However, we observed that dietary *C. butyricum* has no significant effect on the expression of IgA in the intestine. This is similar to the results of other studies, which demonstrate that *C. butyricum* supplementation does not affect the levels of IgA in neonatal mice, broilers, and laying hens (Liao et al., 2015; Miao et al., 2018; Zhan et al., 2019). Perdigon et al. (1995) show that *Lactobacillus casei* and *Lactobacillus acidophilus* improve the level of IgA in a dose-dependent manner. Therefore, we assume that the different effects of *C. butyricum* on the level of IgA may be related to the dose of *C. butyricum*.

Occludin and Claudin are tight junction proteins that play critical roles in regulating intestinal epithelial barrier function and preventing macromolecular transmission (Ballard et al., 1995; Fanning et al., 1998). We found that *C. perfringens* and a high-fishmeal diet decrease levels of Claudin-1 without influencing the expression of Occludin and Claudin-2, which is contrary to the report by Liu et al. (2012). However, *C. butyricum* supplementation was found to elevate the expression of Claudin-1 in *C. perfringens*-infected chickens. This finding suggests that *C. butyricum* may ameliorate damage to the intestinal barrier induced by *C. perfringens*.

In addition, we found that dietary *C. butyricum* could decrease the abundance of *C. perfringens* in the intestine of challenged chickens, consistent with previous reports that probiotics can eradicate pathogens (Gaggia et al., 2010; Yang et al., 2012). However, *C. butyricum* did not have any effect on intestinal lesion.

With regard to the alpha diversity of the gut microbiota, we observed that during the basal diet stage, *C. butyricum* had no significant effect on the ACE and Simpson indices, which is consistent with previous reports that the alpha diversity of intestinal bacteria is not affected by *C. butyricum* (Zhang et al., 2018). However, in the high-fishmeal-supplementation groups, *C. butyricum* supplementation was found to increase the Simpson indices, indicating that *C. butyricum* could reduce the diversity of intestinal bacteria. We speculate that high-fishmeal diets provide an opportunity for the proliferation of *C. butyricum*, which then competes with other bacteria for nutrients and niches and hence reduces the microbial diversity of the chicken intestine under a high-fishmeal diet. In addition, *C. perfringens* causes a decrease in the ACE index, which cannot be improved with *C. butyricum* supplementation.

The results of the PCoA indicate that in the uninfected groups, *C. butyricum* supplementation significantly alters the structure of intestinal microbiota, independent of a high-fishmeal diet, which is consistent with the results of a previous study (Kong et al., 2011). Nevertheless, no significant difference was found between the PCoA results of the *C. butyricum* supplemented and non-supplemented groups after infection. This indicates that *C. butyricum* is unable to alter the disruption of the gut bacterial structure caused by *C. perfringens* infection.

In the basal diet groups, dietary *C. butyricum* could decrease the abundance of harmful bacteria, such as *Candidatus Arthromitus* and unclassified_g__*Brachybacterium*, and increase the abundance of beneficial bacteria, such as *Lactobacillus*_sp._KC45b. *Candidatus Arthromitus*, one group of segmented filamentous bacteria (SFB), has been found in the intestinal bacteria of vertebrates such as chickens, rodents, and fish. It has been reported that the presence of SFB is correlated with diarrhea in poultry (Goodwin and Krabill, 1989; Angel et al., 1990; Morishita et al., 1992). *Brachybacterium* spp. were first isolated from poultry deep litter (Collins et al., 1988) and have previously been considered non-harmful bacteria. However, a recent study shows that *Brachybacterium* spp. are associated with blood infections in humans (Tamai et al., 2018). *Lactobacillus* spp. exerts a beneficial effect of resistance to pathogens such as *Campylobacter* (Stern et al., 2001) and *Clostridium* populations (Decroos et al., 2004). This result is consistent with previous studies that indicate that *C. butyricum* benefits the composition of intestinal bacteria (Yang et al., 2012). However, similar results were not found in the high-fishmeal-supplemented groups, indicating that although a high-fishmeal diet could facilitate the abundance of *C. butyricum* compared with that of basal diet groups, the structure and composition of intestinal bacteria in high-fishmeal-supplemented chickens is not better than that of basal diet chickens.

*C. perfringens* can disrupt the balance of chicken intestinal bacteria. For example, we observed that *C. perfringens* challenge decreases the abundance of the beneficial bacterium *Lactobacillus salivarius*, and this finding is consistent with those of previous studies (Lin et al., 2017; Huang et al., 2018). The results of the PCoA and species composition show that there are no significant differences between the intestinal microbiota structures in the CB/CP and CP groups, indicating that the beneficial effect of *C. butyricum* is less than the harmful effect of *C. perfringens* on the structure of the intestinal flora. However, compared with the CK group, the abundance of beneficial bacteria *Weissella thailandensis*, unclassified_g__*Weissella*, and *Pediococcus acidilactici* increased in the CB/CP group. *Weissella thailandensis*, a class of lactic acid bacteria (LAB), are most commonly used as probiotics and play key roles in disease resistance (Rolfe, 2000). Previous studies have shown that *Pediococcus acidilactici* can act as a potential probiotic as it produces lactic acid and bacteriocins against other enteric pathogens (Daeschel and Klaenhammer, 1985; Klaenhammer, 1993). These results suggest that *C. butyricum* has a certain beneficial effect on the intestinal microbiota of NE chickens.

It has been reported that the probiotics *Lactobacillus acidophilus* and *Bacillus coagulans* (Li Z. et al., 2018; Wu et al., 2018) significantly decrease the lesion score and have a significant beneficial influence on the intestinal bacteria of NE chickens. It has also been demonstrated that *C. butyricum* exerts preventive and therapeutic effects on bacterial infections such as enterohemorrhagic *Escherichia coli*, *Clostridium difficile*, and *Salmonella enteritidis* (Takahashi et al., 2004; Zhao et al., 2017; Oka et al., 2018). However, another study reported that *C. butyricum* could not prevent *Salmonella typhimurium* infection (Peeters et al., 2019). In line with this finding, we observed that *C. butyricum* has no significant effect on the lesion score and the structure of intestinal microbiota after *C. perfringens* challenge. The reason we speculate is that *C. butyricum* could not improve the gut microbiota disturbance induced by NE. In other words, the beneficial effect of *C. butyricum* on the structure of intestinal bacteria is not as strong as the harmful effect of NE. In addition, the dose of *C. butyricum* also has an effect. Different doses of *C. butyricum* have different influences on the expression of intestinal barrier function and inflammatory genes. High-dose *C. butyricum* can promote the expression of Claudin and Occludin, whereas low-dose *C. butyricum* decreases the mRNA levels of these genes (Liu et al., 2018). Symbiotic products and the time-point at which administration takes place also affect its efficacy against NE in chicken. In general, mixed products, such as probiotics and prebiotics, have a greater influence than individual components alone. To the best of our knowledge, probiotics, such as *Bifidobacterium*, *Bacillus subtilis*, *Bacillus licheniformis*, *Lactobacillus salivarius*, and *Lactobacillus plantarum* (Murry et al., 2004; Teo and Tan, 2005; Schoster et al., 2013; Lin et al., 2017), and prebiotics, such as chicory fructans, can inhibit *C. perfringens* growth (Kleessen et al., 2003). In addition, the time-point at which it is administered is also important for probiotic effectiveness. Nakphaichit et al. (2011) reported that dietary *Lactobacillus reuteri* supplementation during the first week of life has a beneficial effect on the composition of intestinal bacteria and that the effects last for up to 6 weeks. Therefore, it is difficult to predict the specific reason for the failure of the CB treatment based only on this single experimental trial. Therefore, further studies will be conducted to find a more reasonable scheme of administration of probiotics to prevent and treat NE in chickens.

In conclusion, *C. butyricum* can inhibit the increase of IL-17A gene expression and the decrease of Claudin-1 gene expression induced by *C. perfringens* challenge. Moreover, dietary *C. butyricum* promotes the expression of IL-10 in infected chickens. This finding suggests that *C. butyricum* has a beneficial effect on the immune response and intestinal barrier function of NE chickens. *C. butyricum* can significantly shift the structure and composition of intestinal bacteria during the stages of basal diet and high-fishmeal-supplementation diet. *C. butyricum* has a particularly large beneficial effect on the structure of intestinal bacteria during the basal diet stage. Meanwhile, *C. butyricum* decreases the abundance of *C. perfringens* in the gut. However, *C. butyricum* cannot significantly alter the structure of intestinal microbiota in *C. perfringens-*challenged chickens and does not have a significant effect on the occurrence of intestinal lesions induced by NE. Therefore, schemes that are more effective than *C. butyricum* supplementation need to be identified to prevent and treat NE in chickens.

## Data Availability Statement

The datasets generated for this study can be found in Sequence Read Archive database, SRP159781.

## Ethics Statement

This study was carried out in accordance with the principles of the Basel Declaration and recommendations of the Institutional Animal Care and Use Committee guidelines, Guangdong Academy of Agricultural Sciences. The protocol was approved by the Guangdong Academy of Agricultural Sciences (No. SYXK (yue)-20180108).

## AUTHOR CONTRIBUTIONS

X-YP and Z-HX conceived the study and participated in its design and coordination. TH designed the experiments and drafted the manuscript. TH, BG, Q-LW, M-GY, and RX carried out the animal experiments. TH and BG carried out the amplicon sequencing and qPCR. TH participated in the statistical analyses. All authors read and approved the final manuscript.

## Conflict of Interest

The authors declare that the research was conducted in the absence of any commercial or financial relationships that could be construed as a potential conflict of interest.

## Footnotes

1 http://drive5.com/uparse/

2 http://rdp.cme.msu.edu/classifier/classifier.jsp

## Supplementary Material

FIGURE S1 Maximum-likelihood tree based on 16S rRNA gene sequences showing the phylogenetic relationships between strain *C. butyricum* YH 018 and closely related species of the genus *Clostridium*. The tree was constructed using the program MEGA 6 with bootstrap values based on 1000 replications. Filled circles mark nodes that were also present in respective maximum-parsimony and neighbor-joining trees. Scale bar, 0.01 nucleotide substitutions per position.

FIGURE S2 Shifts in gut bacterial composition in different groups at order level. ^∗^*p* < 0.05 measured using the Kruskal-Wallis test. CB_B, CK_B, CB_FM, and CK_FM: *n* = 12; CB/CP, CB, CP, and CK: *n* = 6.

TABLE S1 Composition and nutritive levels of the diets (air-dry basis, %).

TABLE S2 Primers used for qPCR.

TABLE S3 Relative intestinal microbiota abundances (%) of significantly different species on three different stage of chicken with NE.
