# Supplementary figures and images for: The Effect of Clostridium butyricum on Gut Microbiota, Immune Response and Intestinal Barrier Function During the Development of Necrotic Enteritis in Chickens
Source: Front Microbiol. 2019 Oct 11;10:2309. doi: 10.3389/fmicb.2019.02309 (PMC6797560; doi:10.3389/fmicb.2019.02309)

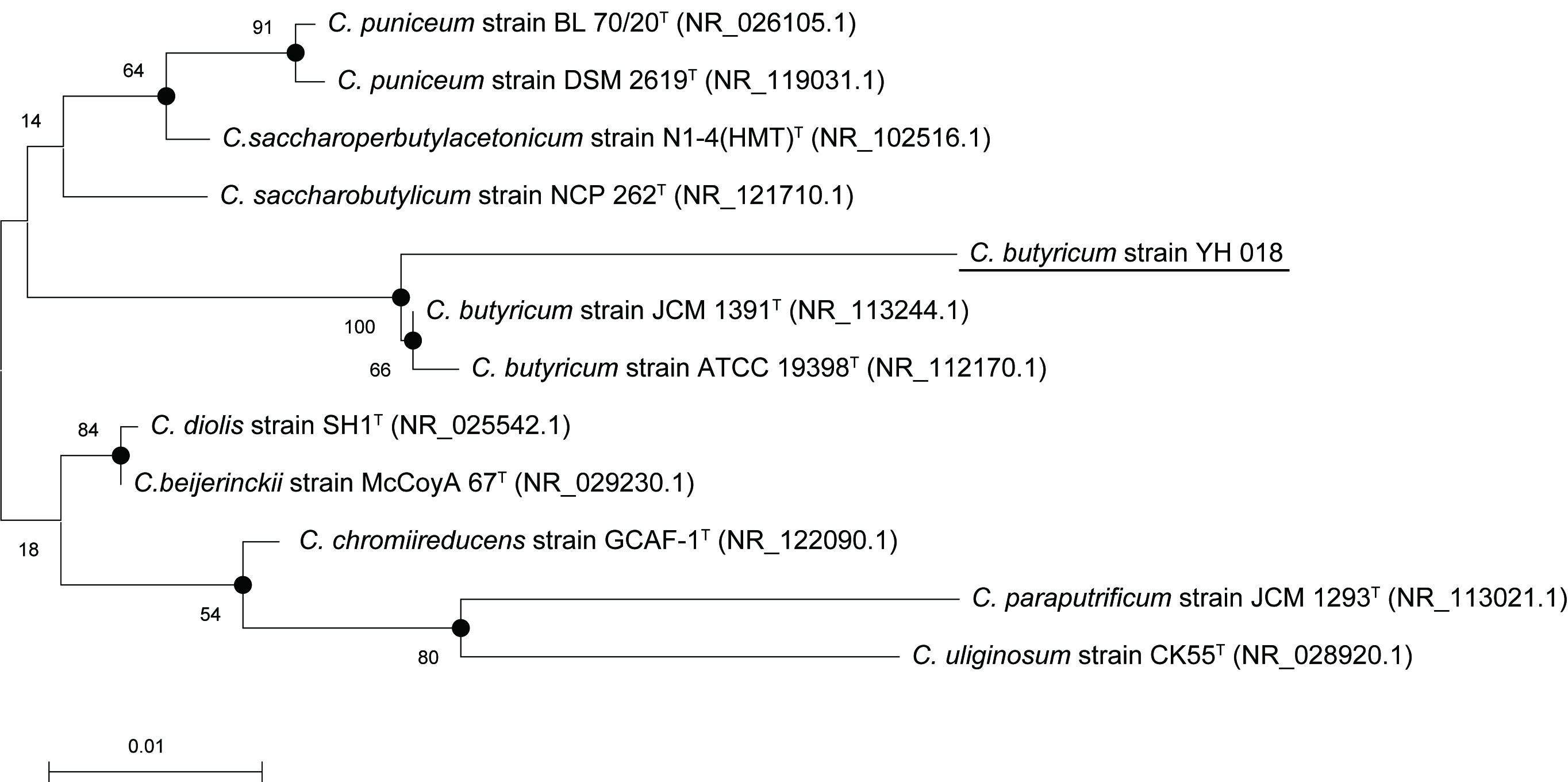

Supplement: FIGURE S1 — Maximum-likelihood tree based on 16S rRNA gene sequences showing the phylogenetic relationships between strain C. butyricum YH 018 and closely related species of the genus Clostridium. The tree was constructed using the program MEGA 6 with bootstrap values based on 1000 replications. Filled circles mark nodes that were also present in respective maximum-parsimony and neighbor-joining trees. Scale bar, 0.01 nucleotide substitutions per position. [file Image_1.TIF]

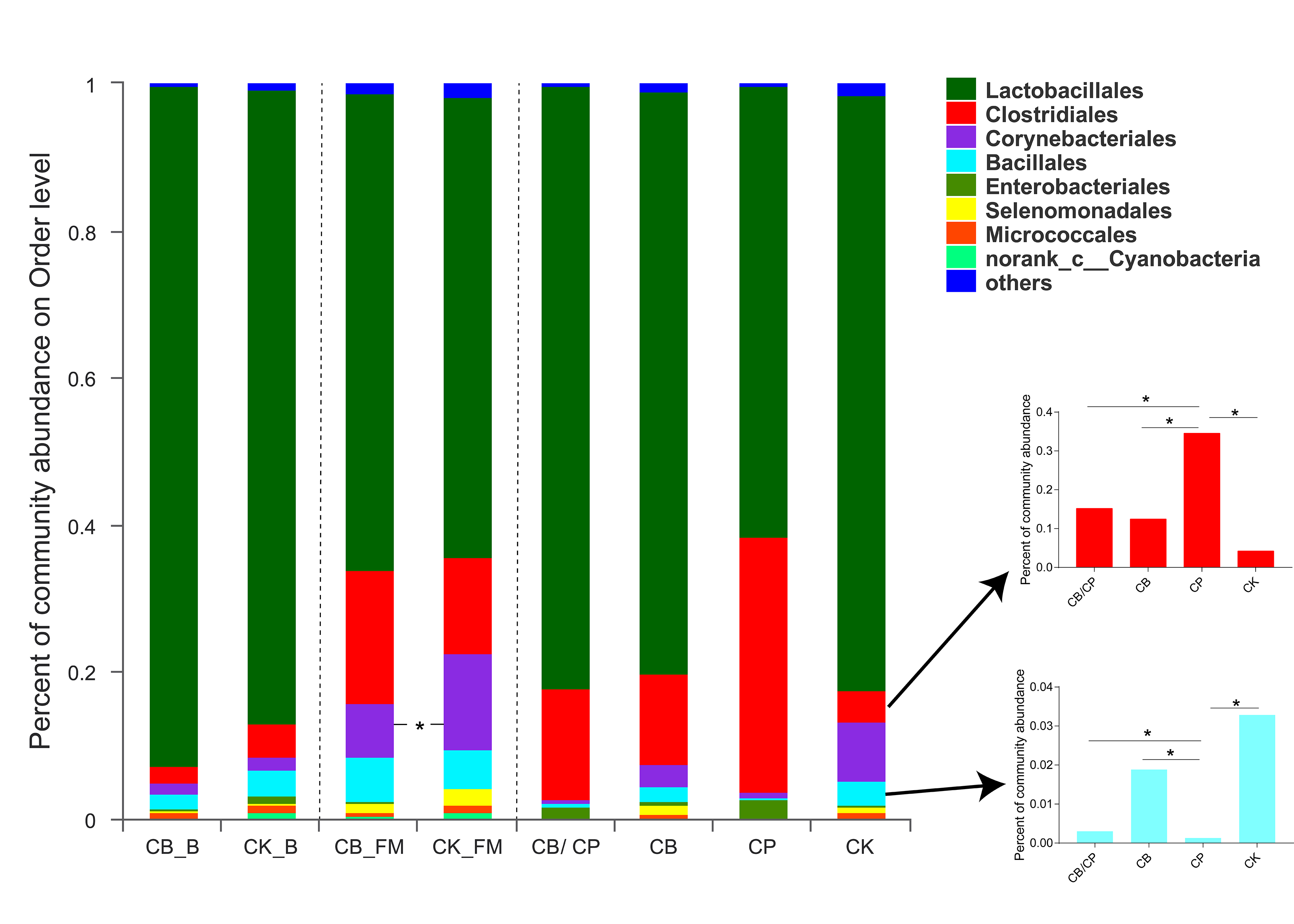

Supplement: FIGURE S2 — Shifts in gut bacterial composition in different groups at order level. ∗p < 0.05 measured using the Kruskal-Wallis test. CB_B, CK_B, CB_FM, and CK_FM: n = 12; CB/CP, CB, CP, and CK: n = 6. [file Image_2.TIF]
