# Supplementary material for: The Effect of Clostridium butyricum on Gut Microbiota, Immune Response and Intestinal Barrier Function During the Development of Necrotic Enteritis in Chickens
Source: Front Microbiol. 2019 Oct 11;10:2309. doi: 10.3389/fmicb.2019.02309 (PMC6797560; doi:10.3389/fmicb.2019.02309)
Supplement: TABLE S2 — Primers used for qPCR. [file Table_2.DOCX]

Table S2 Primers used for qPCR

| Target gene | Forward sequence (5’ to 3’) | Reverse sequence (5’ to 3’) | Reference |
| --- | --- | --- | --- |
| TLR2 | GGGGCTTCACTTCTCTGCTT | AGCATCCTCTGAGATTTGACG | (Monk et al., 2015) |
| IL-10 | GGTTGCCAAGCCTTATCGGA | ACCTGCTCCACTGCCTTGCT |  |
| TNF | TGCTGTTCTATGACCGCC | CTTTCAGAGCATCAACGCA | (Zhao et al., 2017) |
| IL-1β | GTGAGGCTCAACATTGCGCTGTA | TGTCCAGGCGGTAGAAGATGAAG |  |
| NF-kB | CAGCCCATCTATGACAACCG | TCCCTGCGTCTCCTCTGTGA |  |
| IL-17A | CTCCGATCCCTTATTCTCCTC | AAGCGGTTGTGGTCCTCAT | (Lee et al., 2013) |
| Claudin-1 | CATACTCCTGGGTCTGGTTGGT | GACAGCCATCCGCATCTTCT | (Zhang et al., 2017) |
| Claudin-2 | CCATGGTCTCTATGGGACTCC | GCTTCTACACGTATCCCGTC |  |
| Occduin1 | ACGGCAGCACCTACCTCAA | GGGCGAAGAAGCAGATGAG |  |
| GAPDH | TGCTGCCCAGAACATCATCC | ACGGCAGGTCAGGTCAACAA |  |
| IgA | CGTCCAAGAATTGGATGTGA | AGTGACAGGCTGGGATGG | (Monk et al., 2016) |
| *C. perfringen*s | GGCGGTAATATATCTGTTGAAGG | ACCGTCCTTAGTCTCAAC | (Schlegel et al., 2012) |
| 16s | GCCAGCAGCCGCGGTAA | AGGGTATCTAATCCT | (Buffie et al., 2015) |

IL, interleukin; TNF-α, tumour necrosis factor α; TLR, Toll-like receptor; GAPDH, glyceraldehyde-3-phosphate dehydrogenase.

**Reference**

Buffie, C.G., Bucci, V., Stein, R.R., Mckenney, P.T., Ling, L., Gobourne, A., No, D., Liu, H., Kinnebrew, M., Viale, A., Littmann, E., Van Den Brink, M.R., Jenq, R.R., Taur, Y., Sander, C., Cross, J.R., Toussaint, N.C., Xavier, J.B., and Pamer, E.G. (2015). Precision microbiome reconstitution restores bile acid mediated resistance to *Clostridium difficile*. *Nature* 517**,** 205-208. Doi: 10.1038/nature13828

Lee, S.H., Lillehoj, H.S., Jang, S.I., Lillehoj, E.P., Min, W., and Bravo, D.M. (2013). Dietary supplementation of young broiler chickens with Capsicum and turmeric oleoresins increases resistance to necrotic enteritis. *Br J Nutr* 110**,** 840-847. Doi: 10.1017/S0007114512006083

Monk, J.M., Lepp, D., Zhang, C.P., Wu, W., Zarepoor, L., Lu, J.T., Pauls, K.P., Tsao, R., Wood, G.A., Robinson, L.E., and Power, K.A. (2016). Diets enriched with cranberry beans alter the microbiota and mitigate colitis severity and associated inflammation. J Nutr Biochem 28, 129-139. Doi: 10.1016/j.jnutbio.2015.10.014

Monk, J.M., Zhang, C.P., Wu, W., Zarepoor, L., Lu, J.T., Liu, R., Pauls, K.P., Wood, G.A., Tsao, R., Robinson, L.E., and Power, K.A. (2015). White and dark kidney beans reduce colonic mucosal damage and inflammation in response to dextran sodium sulfate. *J Nutr Biochem* 26**,** 752-760. Doi: 10.1016/j.jnutbio.2015.02.003

Schlegel, B.J., Nowell, V.J., Parreira, V.R., Soltes, G., and Prescott, J.F. (2012). Toxin-associated and other genes in *Clostridium perfringens* type A isolates from bovine clostridial abomasitis (BCA) and jejunal hemorrhage syndrome (JHS). *Can J Vet Res* 76**,** 248-254.

Zhang, B., Lv, Z., Li, H., Guo, S., Liu, D., and Guo, Y. (2017). Dietary l-arginine inhibits intestinal *Clostridium perfringens* colonisation and attenuates intestinal mucosal injury in broiler chickens. *Br J Nutr* 118**,** 321-332. Doi: 10.1017/S0007114517002094

Zhao, X., Yang, J., Wang, L., Lin, H., and Sun, S. (2017). Protection Mechanism of *Clostridium butyricum* against Salmonella Enteritidis Infection in Broilers. *Front Microbiol* 8**,** 1523. Doi: 10.3389/fmicb.2017.01523
