# Supplementary material for: The Effect of Clostridium butyricum on Gut Microbiota, Immune Response and Intestinal Barrier Function During the Development of Necrotic Enteritis in Chickens
Source: Front Microbiol. 2019 Oct 11;10:2309. doi: 10.3389/fmicb.2019.02309 (PMC6797560; doi:10.3389/fmicb.2019.02309)
Supplement: TABLE S3 — Relative intestinal microbiota abundances (%) of significantly different species on three different stage of chicken with NE. [file Table_3.DOCX]

Table S3 Relative intestinal microbiota abundances (%) of significantly different species on three different stage of chicken with NE.

| Stage | Classification levels of bacteria | | | |  |  |  |  |
| --- | --- | --- | --- | --- | --- | --- | --- | --- |
|  | Phylum | Order | Family | Species |  |  |  |  |
| Basal diet |  |  |  |  | CB_B | CK_B | *p*-value | FDR |
|  | Firmicutes | Clostridiales | Clostridiaceae | Clostridium_butyricum | 0.3818 | 0.0256 | 0.0002 | 0.0661 |
|  |  |  |  | uncultured_bacterium_g__Candidatus_Arthromitus | 0.0524 | 3.2340 | 0.0013 | 0.0956 |
|  |  | Lactobacillales | Lactobacillaceae | Lactobacillus_sp._KC45b | 0.2076 | 0.0073 | 0.0014 | 0.0956 |
|  | Actinobacteria | Micrococcales | Dermabacteraceae | unclassified_g__Brachybacterium | 0.0025 | 0.0221 | 0.0006 | 0.0877 |
|  | Deinococcus Thermus | Deinococcales | Deinococcaceae | unclassified_g__Deinococcus | 0.0003 | 0.0174 | 0.0008 | 0.0877 |
| High fishmeal supplement |  |  |  |  | CB_FM | CK_FM | *p*-value | FDR |
|  | Firmicutes | Clostridiales | Clostridiaceae | Clostridium_butyricum | 10.7600 | 0.0177 | 0.0000 | 0.0166 |
|  |  |  |  | unclassified_g__Clostridium_sensu_stricto_13 | 0.4398 | 0.0256 | 0.0001 | 0.0175 |
|  |  |  |  | unclassified_f__Clostridiaceae_1 | 0.0902 | 0.0104 | 0.0004 | 0.0489 |
|  |  |  | Peptostreptococcaceae | unclassified_g__Peptostreptococcus | 0.9998 | 0.0716 | 0.0006 | 0.0563 |
|  |  | Bacillales | Bacillaceae | Bacillus_galactosidilyticus | 0.0000 | 0.0164 | 0.0011 | 0.0849 |
|  |  | Lactobacillales | Streptococcaceae | Lactococcus_raffinolactis | 0.1451 | 0.4423 | 0.0004 | 0.0489 |
| *C. perfringens* challenge |  |  |  |  | CB/CP | CK | *p*-value | FDR |
|  | Firmicutes | Bacillales | Staphylococcaceae | uncultured_bacterium_g__Jeotgalicoccus | 0.0006 | 0.0252 | 0.0036 | 0.0931 |
|  |  |  |  | Macrococcus_caseolyticus | 0.0360 | 0.6102 | 0.0081 | 0.0989 |
|  |  |  | Bacillaceae | unclassified_g__Bacillus | 0.0145 | 0.5755 | 0.0081 | 0.0989 |
|  |  | Clostridiales | Peptostreptococcaceae | unclassified_g__Terrisporobacter | 0.0006 | 0.0322 | 0.0057 | 0.0984 |
|  |  |  |  | unclassified_f__Peptostreptococcaceae | 0.0095 | 0.4467 | 0.0081 | 0.0989 |
|  |  |  |  | unclassified_g__Paraclostridium | 0.0006 | 0.0417 | 0.0046 | 0.0931 |
|  |  |  | Clostridiaceae | Clostridium_butyricum | 0.3161 | 0.0183 | 0.0050 | 0.0931 |
|  |  |  | Family_XI_o__Clostridiales | unclassified_g__Tepidimicrobium | 0.0013 | 0.0290 | 0.0066 | 0.0989 |
|  |  |  | Lachnospiraceae | uncultured_bacterium_g__Blautia | 0.0013 | 0.0202 | 0.0047 | 0.0931 |
|  |  |  |  | uncultured_Clostridiales_bacterium_g__[Ruminococcus]_torques_group | 0.0013 | 0.2574 | 0.0078 | 0.0989 |
|  |  | Lactobacillales | Lactobacillaceae | uncultured_compost_bacterium_g__Lactobacillus | 0.4240 | 0.0000 | 0.0028 | 0.0931 |
|  |  |  |  | Pediococcus_acidilactici_g__Pediococcus | 13.0400 | 0.0063 | 0.0047 | 0.0931 |
|  |  |  |  | Lactobacillus_plantarum_subsp._plantarum | 0.0473 | 0.0032 | 0.0077 | 0.0989 |
|  |  |  |  | Lactobacillus_salivarius | 5.4830 | 40.4300 | 0.0082 | 0.0989 |
|  |  |  |  | Lactobacillus_crustorum | 6.2510 | 0.0050 | 0.0047 | 0.0931 |
|  |  |  | Streptococcaceae | Streptococcus_uberis | 0.1508 | 4.5790 | 0.0082 | 0.0989 |
|  |  |  |  | unclassified_g__Lactococcus | 0.0120 | 1.2710 | 0.0048 | 0.0931 |
|  |  |  | Leuconostocaceae | unclassified_g__Weissella | 32.8800 | 0.0618 | 0.0051 | 0.0931 |
|  |  |  |  | Weissella_thailandensis | 9.3960 | 0.0120 | 0.0051 | 0.0931 |
|  |  | Erysipelotrichales | Erysipelotrichaceae | [Clostridium]_spiroforme | 0.0000 | 0.0820 | 0.0026 | 0.0931 |
|  | Actinobacteria | Corynebacteriales | Corynebacteriaceae | unclassified_g__Corynebacterium_1 | 0.3748 | 7.3890 | 0.0050 | 0.0931 |
|  |  | Coriobacteriales | Coriobacteriaceae | unclassified_g__norank_f__Coriobacteriaceae | 0.0000 | 0.0107 | 0.0025 | 0.0931 |
|  |  | Micrococcales | Dermabacteraceae | unclassified_g__Dermabacter | 0.0013 | 0.1066 | 0.0047 | 0.0931 |
|  |  |  |  | unclassified_g__Brachybacterium | 0.0019 | 0.1111 | 0.0043 | 0.0931 |
|  |  | Propionibacteriales | Propionibacteriaceae | unclassified_g__Luteococcus | 0.0063 | 0.2732 | 0.0043 | 0.0931 |
|  | Proteobacteria | Alteromonadales | Shewanellaceae | Shewanella_putrefaciens_CN-32 | 0.0006 | 0.0511 | 0.0047 | 0.0931 |
|  |  |  |  |  | CP | CK | *p*-value | FDR |
|  | Firmicutes | Bacillales | Staphylococcaceae | uncultured_bacterium_g__Jeotgalicoccus | 0 | 0.0252 | 0.0027 | 0.0922 |
|  |  |  |  | Macrococcus_caseolyticus | 0.0158 | 0.6102 | 0.0050 | 0.0922 |
|  |  | Clostridiales | Clostridiaceae | uncultured_bacterium_g__Proteiniclasticum | 0.0076 | 0.3622 | 0.0049 | 0.0922 |
|  |  |  | Family_XI_o__Clostridiales | unclassified_g__Tepidimicrobium | 0.0006 | 0.029 | 0.0046 | 0.0922 |
|  |  |  | Lachnospiraceae | uncultured_bacterium_g__Blautia | 0.0006 | 0.0202 | 0.0037 | 0.0922 |
|  |  |  | Peptostreptococcaceae | unclassified_g__Paraclostridium | 0 | 0.0417 | 0.0027 | 0.0922 |
|  |  |  |  | unclassified_f__Peptostreptococcaceae | 0.0025 | 0.4467 | 0.0047 | 0.0922 |
|  |  |  |  | unclassified_g__Peptostreptococcus | 0.0069 | 0.2089 | 0.0048 | 0.0922 |
|  |  | Erysipelotrichales | Erysipelotrichaceae | [Clostridium]_spiroforme | 0 | 0.082 | 0.0026 | 0.0922 |
|  |  | Lactobacillales | Enterococcaceae | unclassified_g__Enterococcus | 0.0101 | 0.1748 | 0.0047 | 0.0922 |
|  |  |  | Lactobacillaceae | Lactobacillus_salivarius | 3.681 | 40.43 | 0.0051 | 0.0922 |
|  |  |  | Streptococcaceae | Streptococcus_uberis | 0.077 | 4.579 | 0.0050 | 0.0922 |
|  |  |  |  | Streptococcus_parauberis | 0.0082 | 0.212 | 0.0050 | 0.0922 |
|  |  |  |  | unclassified_g__Lactococcus | 0.0095 | 1.271 | 0.0051 | 0.0922 |
|  | Actinobacteria | Coriobacteriales | Coriobacteriaceae | unclassified_g__norank_f__Coriobacteriaceae | 0 | 0.0107 | 0.0025 | 0.0922 |
|  |  | Propionibacteriales | Propionibacteriaceae | unclassified_g__Luteococcus | 0.0114 | 0.2732 | 0.0049 | 0.0922 |
|  | Proteobacteria | Pseudomonadales | Pseudomonadaceae | unclassified_g__Pseudomonas | 0.0006 | 0.0852 | 0.0046 | 0.0922 |

Only those species that are significant difference (*p* < 0.05, FDR< 0.1) are shown. There was no significant difference between the group CB/CP and CP and the group CB and CK. Values are means. CB_B, CK_B, CB_FM and CK_FM: n=12; CB/CP, CP and CK: n=6
